# Intensity correlation scan (IC-scan) technique to characterize the optical nonlinearities of scattering media

**DOI:** 10.1038/s41598-023-34486-0

**Published:** 2023-05-04

**Authors:** Mariana J. B. Crispim, Cícera C. S. Pereira, Nathália T. C. Oliveira, Martine Chevrollier, Rafael A. de Oliveira, Weliton S. Martins, Albert S. Reyna

**Affiliations:** 1 Programa de Pós-Graduação em Engenharia Física, Unidade Acadêmica do Cabo de Santo Agostinho, Universidade Federal Rural de Pernambuco, Cabo de Santo Agostinho, Pernambuco 54518-430 Brazil; 2 Programa de Pós-Graduação em Ciência de Materiais, Universidade Federal de Pernambuco, Recife, Pernambuco 50740-560 Brazil

**Keywords:** Optical spectroscopy, Nonlinear optics

## Abstract

Light scattering, whether caused by desired or spurious elements, is considered one of the main phenomena that present great challenges for the nonlinear (NL) optical characterization of turbid media. The most relevant disturbing factor is the random deformation suffered by the spatial intensity distribution of the laser beam due to multiple scattering. In this work, we report the intensity correlation scan (IC-scan) technique as a new tool to characterize the NL optical response of scattering media, by taking advantage of light scattering to generate speckle patterns sensitive to wavefront changes induced by the self-focusing and self-defocusing effects. Peak-to-valley transmittance curves, with a higher signal-to-noise ratio, are obtained by analyzing the spatial intensity correlation functions of the different speckle patterns, even in very turbid media where conventional NL spectroscopy techniques fail. To demonstrate the potential of the IC-scan technique, the NL characterization of colloids that contain a high concentration of silica nanospheres as scatterers, as well as gold nanorods, which act as NL particles and light scatterers, was performed. The results show that the IC-scan technique is more accurate, precise and robust to measure NL refractive indices in turbid media, overcoming limitations imposed by well-established Z-scan and D4σ techniques.

## Introduction

Light scattering is one of the most fundamental optical phenomena observed due to the interaction of light with matter, resulting from inhomogeneities in the refractive index over the scattering volume^1^. The relevance of scattering in several hard and soft condensed matter systems is evidenced by the various non-invasive techniques developed to measure particle size and colloidal stability^2^, micro-defect detection^3^, optical-tissue diagnostics^4^, as well as to investigate their applications in optical super-resolution^5^, three-dimensional holography^6^, modern cryptography^7^ and random lasers^8^. Even in this last system, by switching from the single-scattering to the multiple-scattering regime, it was possible to study new light diffusion phenomena, such as the glassy light phase compatible with a replica symmetry breaking^9^ and a Floquet phase^10^ in photonic systems, as well as the Anderson localization of light^11^. Nevertheless, the more dense and disordered the medium that interacts with the light, the more significant the distortion caused by the scattered photons in the spatial and temporal intensity profiles of the transmitted or reflected beams, which are not always desired in optical and photonic systems^12–14^.

Speckle patterns are a clear example of the complex intensity distribution that a coherent beam scattered by a disordered medium, with a high degree of scattering, can undergo. These patterns with randomly distributed intensities and phase are the result of the superposition of many different scattered waves that interfere with effectively random phases^15^. For a long time, speckles were considered just as a noisy phenomenon that contaminates the observation of different physical processes, decreasing the signal-to-noise ratio and consequently limiting the precision and sensitivity of many optical techniques^16–19^. Such an interpretation is reasonable when light scattering is caused by spurious particles, *viz.* dust, or system imperfections^20–22^. However, when the speckles are the result of the inherent disorder of the system, the analysis of their statistical properties, such as intensity correlation function and power spectral density, can yield relevant information about the optical properties of the studied system^23^. Significant progress in the statistical study of speckles patterns has been made in stellar physics^24^, random lasers^25–27^, optical-image processing^28^, optical manipulation^29^, accurate measurements of contour, deformation, vibration and strain on various materials^30^, displacements and deformations of diffuse objects^31^ and biological tissues analysis^32^.

In nonlinear (NL) optical spectroscopy, strong light scattering caused by turbid media has been reported to be a problem for most techniques that measure NL refractive index^33–36^. Among them, the well-established Z-scan technique suffers with the distortion of the transmitted intensity beam profile and wavefront induced in the scattering media, which causes the intensity transmitted by the small aperture (*closed aperture* (*CA*) Z-scan) to show large fluctuations at each step during sample translation. Experiences in highly scattering media, such as colloids containing SiO_2_ nanoparticles (NPs) suspended in acetone, show that the fluctuations in the *CA* Z-scan curve can be larger than the peak-valley transmittance variations, making its characterization unfeasible^34^. Similar experiments show a low signal-to-noise ratio in the CA Z-scan curves when vitreous humor^37^, ammonium dihydrogen phosphate crystals^38^, and liquid crystals^39,40^ were studied. To overcome this limitation in scattering media, some adaptations to existing (or new) techniques have been developed^33–35^. In the spatial domain, the scattered light imaging method (SLIM) was proposed to collect light scattered by the turbid medium, in the direction perpendicular to propagation, to image the evolution of the laser beam diameter along propagation^34^. Here, the NL refractive index is determined by analyzing the divergence angle variations induced by the self-focusing (or self-defocusing) effect, even in a single shot configuration. Thus, unlike NL transmission techniques that suffer with light scattering, SLIM can only be used when scattering is relevant and the samples are thick enough to analyze light propagation within scattering media^34^. In addition to measuring the NL refractive index, SLIM was also recently applied to discriminate the NL extinction due to NL absorption and NL scattering contributions in turbid media^41^.

In the spectral domain, new techniques have also been developed to measure NL phase variations in scattering media by analyzing the frequency spectrum shift of a transmitted laser pulse^33,35^. Spectral analysis, as done in the spectral reshaping technique, has the advantage that, unlike beam shape analysis, it is not affected by linear scattering effects^33^. However, its experimental setup is more complex than the spatial Z-scan and SLIM techniques, since it requires the use of ultrashort pulses spectrally reshaped by an acoustic optical modulator, to make a *hole* in the incidence laser spectrum which will be *filled* when passing through a NL medium, due to self-phase modulation. This *hole-refilling* technique was recently adapted to a simpler experimental apparatus, called the spectral domain Z-scan, which has been used to measure the refractive index of frosted fused-silica slide^35^ and human cornea^42^. Despite the good sensitivity of these NL techniques in the spectral domain, their accuracy depends on how well-defined the temporal beam profile is, as well as on the absence of self-steepening effects that induce laser pulse’s spectral broadening. Therefore, the measurement of the NL refractive index in scattering media continues to be a great challenge for NL optics, and its study is supported by the need to characterize biological media, liquid crystals and other materials that present a high-degree of scattering.

In this work, we present the Intensity Correlation scan (IC-scan) as a new NL optical technique, in the spatial domain, which allows the proper NL optical characterization of strongly scattering media caused by system imperfections, or by the presence of linear or NL scatterers. The IC-scan technique uses light scattering caused by turbid media (or light diffuser) to generate speckle patterns, in the far field, that are sensitive to wavefront changes induced by the self-focusing and self-defocusing effects. By analyzing the intensity self-correlation function of the speckle patterns generated during the sample translation around the focus of a lens, it is possible to obtain curves similar to those of Z-scan, but with lower noise levels, even when the scattering is so strong that it destroys the spatial intensity profile of the transmitted beam. Furthermore, the pure self-phase modulation effects, free from linear scattering contributions, can be obtained by analyzing the intensity cross-correlation function between IC-scan measurements performed in the linear and NL regimes. As a proof of principle, NL refractive index measurements were performed by IC-scan on highly concentrated NL ethanolic (aqueous) colloids containing silica nanospheres (gold nanorods) as light scatterers (NL particles and scatterers), using a titanium-sapphire laser (788 nm, 100 fs, 76 MHz), and the results were compared with the well-established Z-scan^43^ and D4σ^44^ techniques.

## Experimental details

### IC-scan setup

**Figure 1:**
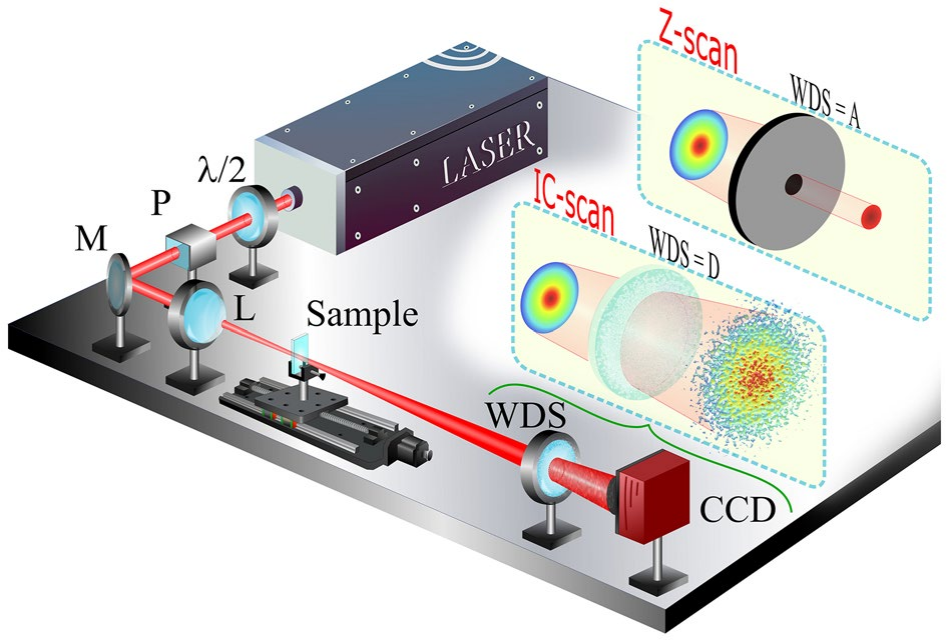
Experimental setup used to characterize the NL response of transparent and turbid media by Z-scan and IC-scan techniques. P: polarizer; M: mirror; L: lens; WDS: wavefront distortion sensor; A: aperture; D: diffuser.

Figure 1 shows the experimental setup used to measure the NL refractive indices of transparent and turbid media. To excite the NL thermal response of the different solvents and colloids, described in the Nonlinear media section, a mode-locked Ti:Sapphire laser emitting 100-fs Gaussian pulses at 788 nm and with a repetition rate of 76 MHz was used. The control of the incident beam’s power was provided by a λ/2 plate followed by a Glan prism (P), which assures that the beam is linearly polarized. Subsequently, the Gaussian beam was focused by a 10-cm focal length lens (L), producing a beam waist of ~ 25.5 μm at the focus position (Rayleigh length: ${z}_{0}\approx 2.6$ mm). For the measurements, a quartz cell (thickness: *L* = 1.0 mm < *z*_0_) filled with NL media, was moved along the beam propagation direction (Z-axis) around the region where the laser beam is focused. The transmitted beam passes through an element sensitive to wavefront distortions (WDS), located in the far-field, and finally its transversal intensity profile is fully recorded by a CCD camera.

The choice of WDS defines the technique that is used to measure the NL optical response of different media. For instance, when the WDS is an iris, the experimental setup corresponds to the well-established *CA* Z-scan technique^43^. On the other hand, when the WDS is removed, the integration of the intensity profile on the CCD, at each step of the NL medium, gives rise to the open aperture (*OA)* Z-scan technique^43^, while the D4σ technique is accessed when the transverse irradiance moments are analyzed^45^. Various types of WDS are reported in the literature as adaptations to the Z-scan technique for measuring NL refractive index (see^46^ and reference therein).

**Figure 2:**
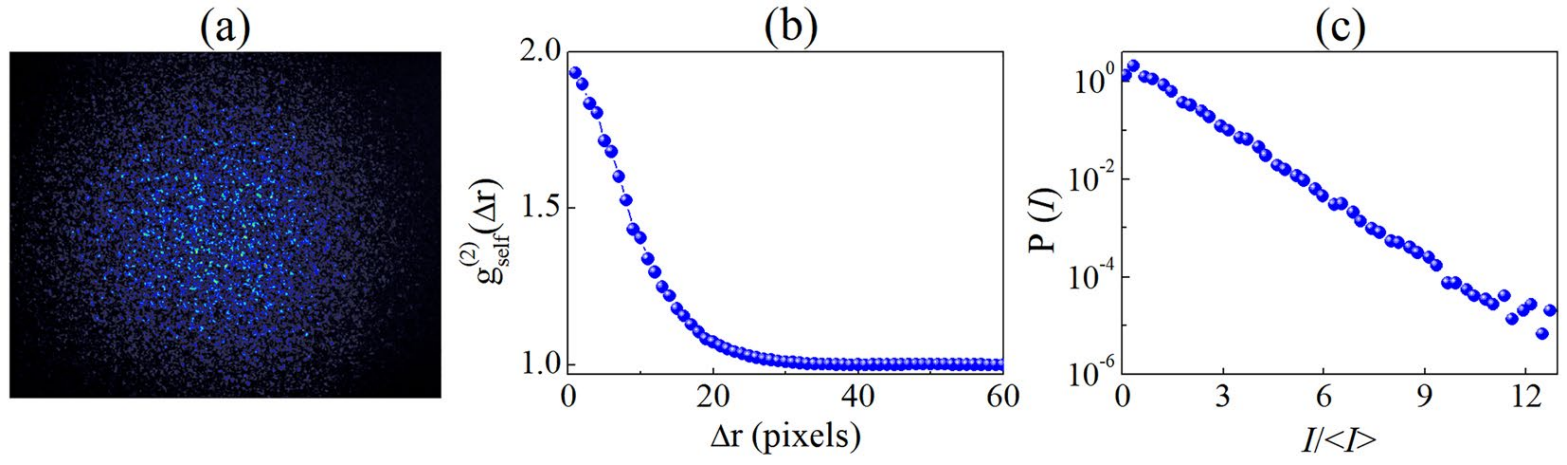
(**a**) Speckle pattern obtained by IC-scan technique and their (**b**) 2D spatial intensity correlation and (**c**) intensity-probability density function. The intensity profile was collected for an empty cell.

The IC-scan technique, proposed in this work, uses transparent light diffusers as WDS to generate speckle patterns, whose intensity correlation function is highly sensitive to wavefront changes induced by self-focusing (or self-defocusing) effects. Figure 2a illustrates a speckle pattern captured by the CCD (1024 × 1280 pixels), when the quartz (sample) cell is empty. The 2D spatial intensity self-correlation function $\left({g}_{self}^{\left(2\right)}\left(\Delta r\right)=\frac{\langle \int {d}^{2}rI\left(r\right)I\left(r+\Delta r\right)\rangle }{\int {d}^{2}r\langle I\left(r\right)\rangle \langle I\left(r+\Delta r\right)\rangle }\right)$, shown in Fig. 2b, exhibits the shape expected for a speckle pattern with Gaussian intensity distribution, varying from ${g}_{self}^{\left(2\right)}\left(0\right)\approx 2.0$ to ${g}_{self}^{\left(2\right)}\left(\infty \right)=1.0$^22^, where the angular brackets $\langle \cdots \rangle$ denote averaging over many realizations. Fifty consecutive images were used to calculate the 2D spatial intensity correlation function at each position of the NL sample. For each image, the laser beam illuminates a different zone of the light diffusor, which is rotated from one image to the next. The width of ${g}_{self}^{\left(2\right)}\left(\Delta r\right)$ gives the mean size of the speckles, which was measured to be ~ 20 pixels. In addition, the normal distribution is corroborated by the intensity-probability density function, $P\left(I\right)$, since it behaves as a function close to a straight line, on a semilogarithmic scale (see Fig. 2c). IC-scan curves are constructed by plotting the maximum value of ${g}_{self}^{\left(2\right)}\left(\Delta r\right)$ as a function of the sample position around the focal plane of the lens *L*, as shown in the numerical simulation section.

It is important to highlight that the experimental setup of the IC-scan technique is very similar to the well-known Z-scan and D4σ techniques. This is because in the three techniques used, the NL phase shift caused in the laser beam that interacts with the NL medium is the result of the refractive index modulation induced by the intensity beam profile, which causes the self-focusing (or self-defocusing) effect. However, the difference between the techniques lies in the detection methodology that varies depending on the type of WDS used. In the IC-scan technique, the analysis of the beam phase is carried out by means of an interferometric measurement introduced by the presence of the diffuser, in the far-field, which gives rises to speckle patterns with different intensity distributions. Thus, the IC-scan technique emerges as an application of speckle metrology dedicated to the measurement of the NL refractive index through the phase deformations monitored by the analysis of the intensity correlation function, which has a solid theoretical basis in several studies^15,47^. We emphasized that the diffusers act only as WDS, i.e., the speckles do not experience the self-focusing (self-defocusing) that usually occurs when they propagate in media with a positive (negative) NL refractive index, as reported in^48^.

### Nonlinear media

**Figure 3:**
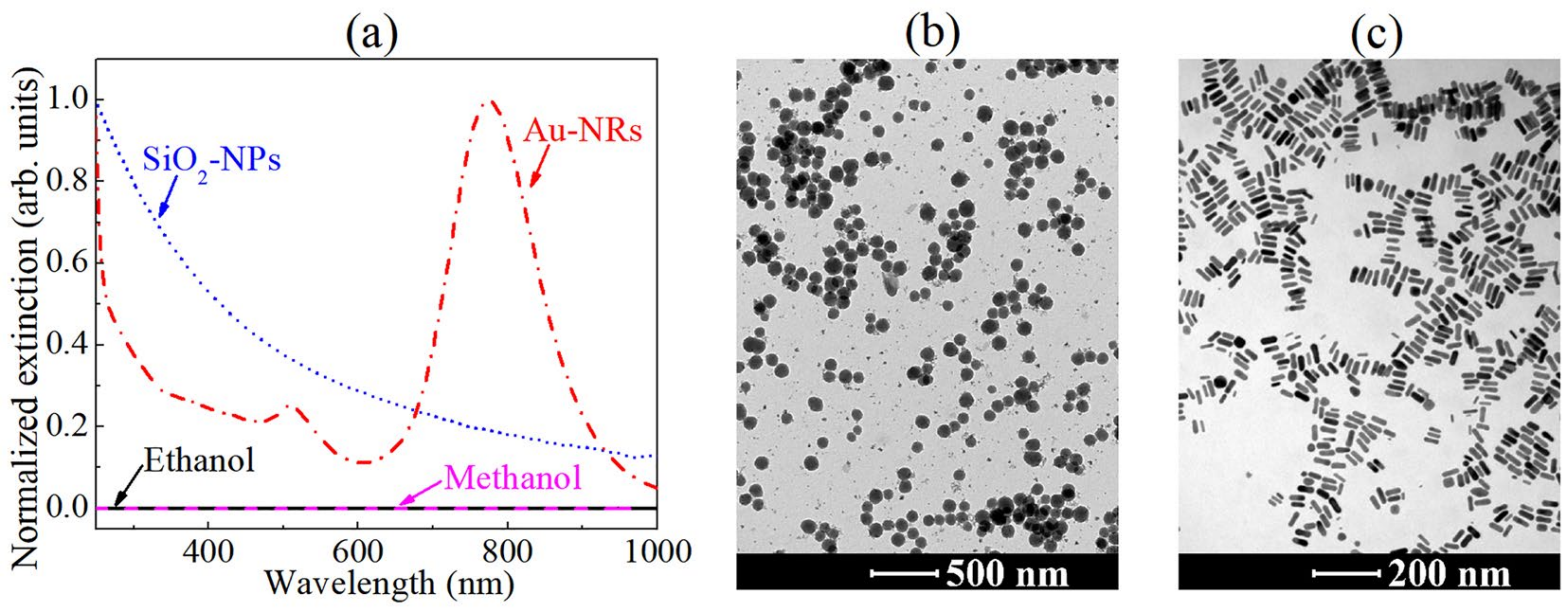
(**a**) Normalized extinction spectra and (**b**,**c**) TEM images for scattering media represented by spherical silica (SiO_2_) NPs and gold nanorods (Au-NRs) suspended in ethanol and water, respectively. The extinction spectra of the solvents were plotted to demonstrate their transparency.

Four NL media, with and without scattering particles, were used to demonstrate the potential of IC-scan to measure the NL refractive indices compared to Z-scan and D4σ techniques. Two of them are typical NL solvents, viz*.* pure ethanol (≥ 99.9%) and methanol (≥ 99.9%), purchased from Sigma-Aldrich, used as transparent (without scatterers) NL media, as shown in Fig. 3a. To represent NL media with different Rayleigh scattering contributions, two colloids containing silica (SiO_2_) NPs suspended in ethanol were prepared, following the procedure described in^49^, with volume fractions of 8.2 × 10^–3^ and 4.1 × 10^–2^. Its transmission electron microscopy (TEM) image (Fig. 3b) reveals spherical particles with an average diameter of ~ 120 nm, and their extinction spectrum is characteristic of Rayleigh scattering because it depends on ${\lambda }^{-4}$, where $\lambda$ is the incident light wavelength.

Colloidal gold nanorods (Au-NRs) were also used as scattering media with strong NL optical behavior. For this, Au-NRs were chemically synthesized by a seed-mediated growth method^50^ to exhibit an average cross-sectional diameter of 15.0 ± 0.6 nm and aspect ratio (AR) equal to 3.4 (see Fig. 3c). The NR’s dimensions were chosen so that its longitudinal localized surface plasmon (*l*-LSP) resonance, which is known to exhibit a significant scattering contribution^51^, is centralized near the laser wavelength (788 nm), as shown in Fig. 3a. For the IC-scan measurements, the Au-NR colloids were diluted in deionized water to present volume fractions of 2.5 × 10^−5^, 5.0 × 10^−5^ and 7.5 × 10^−5^.

## Numerical simulations

### Nonlocal NL response: Z-scan and D4σ

Due to the high repetition rate of the excitation laser, the media studied in this work exhibit an optical nonlinearity dominated by thermal effects. On this origin, the nonlocal model proposed in^52^ was used to simulate the experimental results obtained by the Z-scan, D4σ and IC-scan techniques, because it presented better results than the thermal lens and aberrant thermal lens models. By expressing the optical field in the amplitude-phase form, $E=\left|{E}_{in}\right|\mathrm{exp}\left[i\phi \right]$, the nonlocal model defines that the evolution equations, along the beam propagation axis (z-axis), for the phase and intensity of an incident Gaussian beam passing through a thin NL sample can be written as^53^:

1 $$\frac{{d{\Delta }\phi_{m} }}{dz\prime } = k{\Delta }n\left( {I_{m} } \right)$$

2 $$\frac{d{I}_{m}}{d{z}^{^{\prime}}}=-\alpha \left({I}_{m}\right){I}_{m}$$

where $\Delta n\left({I}_{m}\right)={n}_{2}{I}_{m}={n}_{2}\left({I}_{0}{G}_{m}\right)$ for media exhibiting a third-order refractive index, ${n}_{2}$, and an intensity-dependent extinction coefficient, $\alpha \left({I}_{m}\right)$. ${I}_{m}$ is the nonlocal intensity that can be expressed as the product of the maximum on-axis value, ${I}_{0}$, and a Gaussian nonlocal profile, ${G}_{m}={\left({G}_{local}\right)}^{m/2}$, with ${G}_{local}=\mathrm{exp}\left[-2{r}^{2}/{w\left(z\right)}^{2}\right]{\left[1+{\left(z/{z}_{0}\right)}^{2}\right]}^{-1}$, $w\left(z\right)={w}_{0}\sqrt{1+{\left(z/{z}_{0}\right)}^{2}}$, Gaussian beam waist: $w\left(z=0\right)={w}_{0}$, wavelength: $\lambda$, $k=2\pi /\lambda$ and Rayleigh length: ${z}_{0}$. Notice that the nonlocality factor, $m$, is introduced as a constant that affects the radius of the Gaussian beam in the NL sample^52,53^. For instance, for $m<2$ $\left(m>2\right)$ the NL phase shift extends (compresses) beyond the incident intensity distribution, while for $m=2$ the NL response of the medium is considered as local^43^. It is worth mentioning that the ${n}_{2}$ values measured in this work for $m\ne 2$ are related to the thermo-optic coefficients that tend to induce self-defocusing effects in an equivalent way to the third-order NL refractive indices for the Kerr effect.

To find the amplitude and phase of the optical field in the detection plane, the Fast Fourier beam propagation method (BPM) was used^54^. Numerically, the NL medium of thickness $L$ was divided into $N$ parts of size $\Delta z=L/N$, with the optical field at the end of each step given by $E\left(x,y,z+\Delta z\right)=\widehat{P}\widehat{A}\widehat{L}\widehat{A}\widehat{P}E\left(x,y,z\right)$. The operator $\widehat{P}=\mathrm{exp}\left[ik{n}_{0}\Delta z/2\right]\mathrm{exp}\left\{i\Delta z/2\left[{\nabla }_{t}^{2}/\left(\sqrt{{\nabla }_{t}^{2}+{n}_{0}^{2}{k}^{2}}+{n}_{0}k\right)\right]\right\}$ represents the linear propagation through distance $\Delta z/2$, in a homogeneous medium with linear refractive index ${n}_{0}$ and transverse derivative ${\nabla }_{t}^{2}=-\left({k}_{x}^{2}+{k}_{y}^{2}\right)$ in the Fourier domain. The linear and NL intensity losses along propagation are considered through the operator $\widehat{A}=\mathrm{exp}\left[-\alpha \left({I}_{m}\right)\Delta z/4\right]$, while the operator $\hat{L} = \exp \left[ {ik\mathop \smallint \limits_{z}^{z + \Delta z} \Delta n\left( {I_{m} } \right)dz\prime } \right]$ incorporates the NL phase-shift. Note that $z^{\prime}$ represents the propagation depth in the NL medium and $z$ the sample position around the focus plane. The last two operators, $\widehat{A}$ and $\widehat{P}$ conclude the beam propagation in a step $\Delta z$, where the resulting field will be used as an initial condition for the next step. After sequentially performing *N* iterations, the far-field beam patterns for the different position of the NL sample, around the focal plane, were obtained by numerical simulations on the free-field propagation using the Huygens–Fresnel formalism, following the method described in^54^. Approximate analytical expressions for the nonlocal model were also reported in^53^ using the Gaussian decomposition method.

It is worth mentioning that due to the strong contributions of nonlocal nonlinearities, which induce large NL phase variations (${\Delta \phi }_{0}$), the BPM is important to allow experimental curve fitting considering the thin-sample approximation. That is, in the NL regime, the medium is regarded as “thin”, if the sample length is small enough that changes in the beam diameter within the sample due to nonlinear refraction can be neglected^43^. Since in the BPM method Eqs. (1) and (2) are solved iteratively for sample lengths of a step size, $\Delta z$, the thin-sample criterion implies that $\left(\Delta z=L/N\right)\ll \mathrm{z}_{0}/{\Delta \phi }_{0}$, which is easily obeyed since large values of N were used. In the numerical simulations,* N* was chosen as the minimum value such that when it is duplicated the Z-scan, IC-scan and D4σ curves present the same results. In this work, the minimum value used for N was 1000.

From the simulated far-field beam patterns, ${E}_{F-F}\left(x,y,z\right)$, the Z-scan and D4σ curves were fitted by calculating the normalized transmittance, $T\left(z\right)$, and normalized second-order moments, ${m}_{2}\left(z\right)$, respectively. For Z-scan, the normalized transmittance was calculated by using the expression $T\left(z\right)={\int }_{0}^{{r}_{a}}{\left|{E}_{F-F}\left(x,y,z\right)\right|}^{2}dxdy/{\int }_{0}^{{r}_{a}}{\left|{E}_{F-F}^{\left(0\right)}\left(x,y,z\right)\right|}^{2}dxdy$, where ${r}_{a}$ is the radius of a circular aperture and ${E}_{F-F}^{\left(0\right)}\left(x,y,z\right)$ is the far-field beam pattern when the absorption and refraction NL contributions are zero. To reproduce the experimental conditions of the CA Z-scan scheme, the transmittance was calculated over the area of a circular aperture with a radius of 335 µm (${r}_{a}=$ 50 pixels), centered at $\left({x}_{0},{y}_{0}\right)=\left(\mathrm{0,0}\right)$. The D4σ curves were calculated using the total beam area through the expression: ${m}_{2}\left(z\right)={\iint }_{-\infty }^{\infty }{\left|{E}_{F-F}\left(x,y,z\right)\right|}^{2}{\left(x-\overline{x }\right)}^{2}dxdy/{\iint }_{-\infty }^{\infty }{\left|{E}_{F-F}\left(x,y,z\right)\right|}^{2}dxdy$ with $\overline{x }={\iint }_{-\infty }^{\infty }{\left|{E}_{F-F}\left(x,y,z\right)\right|}^{2}xdxdy/{\iint }_{-\infty }^{\infty }{\left|{E}_{F-F}\left(x,y,z\right)\right|}^{2}dxdy$, and also normalized by ${m}_{2}^{0}\left(z\right)$, calculated when the nonlinearities are zero.

### Light transmission by a diffuser: IC-scan

Since the IC-scan technique uses a light diffuser as an element sensitive to wavefront distortions, the experimental curves were modeled by transmitting far-field beam patterns through a rough surface using the optical transfer function^55^. Briefly, the random fields were generated by multiplying ${E}_{F-F}\left(x,y,z\right)$, obtained in the previous section, by ${e}^{-i\varnothing }$, where $\varnothing (x, y)$ corresponds to a random matrix generated from a uniform distribution in the range of $(-\pi , \pi )$. Thus, the random phase field is given by ${E}_{rand}\left(x, y,z\right)={E}_{F-F}\left(x,y,z\right){e}^{-i\varnothing }$. However, since rough media randomize the field’s phase based on its degree of scattering, a spatial frequency filter was added to reproduce the experimentally obtained speckles patterns. In the spatial frequency domain, we use: $E\left({k}_{x},{k}_{y} ;z\right)={F}_{x,y}[{E}_{rand}\left(x, y;z\right)] H({k}_{x},{k}_{y})$, where the first term denotes the 2D Fourier transform of the ${E}_{rand}\left(x, y;z\right)$ and $H({k}_{x},{k}_{y})$ is a spatial frequency filter consisting of a 4-*f* imaging system containing a mask in the Fourier plane, which allows the transmission of photons whose spatial frequencies pass through a circular aperture of radius *ρ*^56^. Since the spatial frequencies $\left({k}_{x},{k}_{y}\right)$ are mapped in the Fourier plane at the points $\left(x,y\right)=\left(\frac{f{k}_{x}}{k},\frac{f{k}_{y}}{k}\right)$, where *k* corresponds to the wavevector and *f* to the focal length of the lenses used in the 4-*f* system, we have that $H\left({k}_{x},{k}_{y}\right)$ has the same behavior as a pupil function in real space, given by: $h\left(x,y\right)=1$ for coordinates inside a circle of radius *ρ* $\left(\text{i.e. } \sqrt{{x}^{2}{+ y}^{2}}<\rho \right)$ and $h\left(x,y\right)=0$ for otherwise. Thus, $H\left({k}_{x},{k}_{y}\right)$ filters out photons with spatial frequencies (cut-off frequency) lower than $\frac{\rho k}{f}$ $\left(\text{i.e. } \sqrt{{k}_{x}^{2}+{k}_{y}^{2}}<\frac{\rho k}{f}\right)$. Finally, the speckle patterns were obtained by calculating the field in the spatial domain through the inverse Fourier transform, $E\left(x,y,z\right)={F}_{x,y}^{-1}\left[E\left({k}_{x},{k}_{y};z\right)\right]$, followed by free-space propagation to the detection plane, via the split-step BPM^57^.

In this work, we use a circular aperture of radius *ρ* = 2.2 mm to properly simulate the speckle patterns, in the linear regime, as shown by the solid curves in Fig. 2b,c. These values were kept constant for the analysis of the IC-scan curves in the NL regime.

## Results and discussions

### NL refractive optical characterization of transparent media

**Figure 4:**
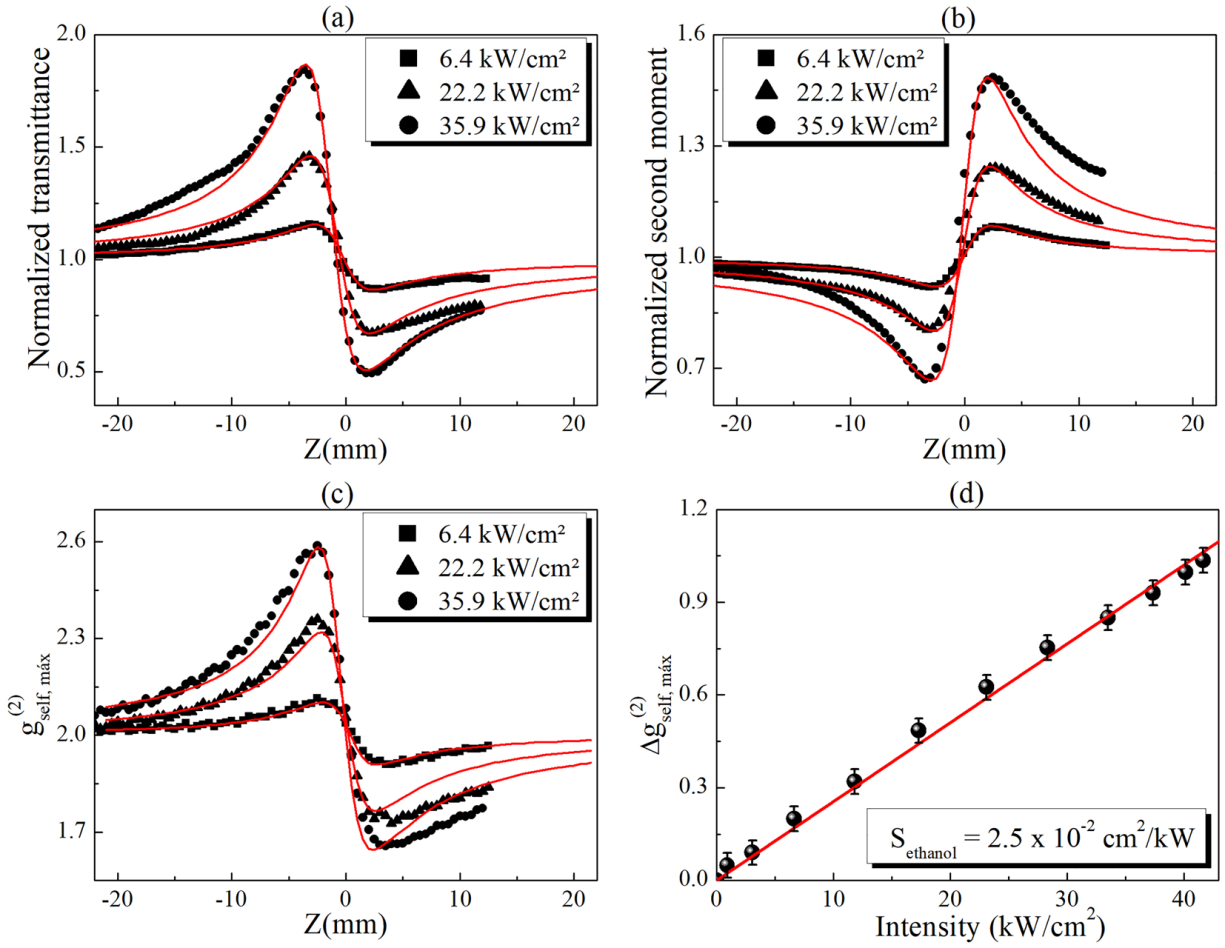
(**a**) Z-scan, (**b**) D4σ and (**c**) IC-scan curves obtained for ethanol at different intensities. The solid lines in (**a**) and (**b**) represent the best curve fits using the nonlocal model (section 3.A), while in (**c**) represent the numerical simulation using the procedure described in section 3.B. (**d**) Peak-to-valley variation of IC-scan curves for ethanol as a function of incident intensity.

Figure 4 shows the experimental results obtained by the CA Z-scan, D4σ and IC-scan techniques performed to characterize the refractive NL response of pure ethanol. A good signal-to-noise ratio is observed in the curves because scattering particles (or defects) are not present in the NL medium. In Fig. 4a,b, by fitting the experimental results using the nonlocal model (with $m=0.1$) described in the Numerical simulation section, the NL refractive indices ${n}_{2}^{ethanol}=-\left(2.8\pm 0.4\right)\times 1{0}^{-8} {\text{cm}}^{2}/{\text{W}}$ (for Z-scan) and $-\left(2.2\pm 0.3\right)\times 1{0}^{-8} {\text{cm}}^{2}/{\text{W}}$ (for D4σ), were obtained. The difference between the values measured by CA Z-scan and D4σ is probably due to the asymmetry or imperfection in the Gaussian beam profile that causes alterations when analyzing the transmittance through a small aperture or the transverse irradiance (second-order) moments, respectively. Nevertheless, reports in the literature using the local NL model $\left(m=2.0\right)$ to fit the experimental curves showed ${n}_{2}$ values lower than those obtained by us (see for example^58^). However, because the thermal contribution is dominant in continuous or quasi-continuous excitation, the nonlocal NL model presents better results when compared to the experimental curves, as shown in Fig. 4. Comparisons between the local and nonlocal models to fit the experimental curves are described in the Supplementary Material.

The IC-scan curves, shown in Fig. 4c, were obtained by calculating the maximum value of ${g}_{self}^{\left(2\right)}\left(\Delta r\right)$, i.e. ${g}_{self, max}^{\left(2\right)}={g}_{self}^{\left(2\right)}\left(0\right)$, as a function of the sample position, when a light diffuser is used as WDS in the far field (5 cm before the CCD). Profiles similar to those of Z-scan, with a peak-valley structure, are observed in the IC-scan curves, starting with ${g}_{self, max}^{\left(2\right)}=2$ in the linear regime and increasing (or decreasing) due to beam divergence angle variations caused by the NL phase shift at high intensities. The variations of ${g}_{self, max}^{\left(2\right)}=2.0$ can be understood from the different intensity distributions that the speckle patterns exhibit when a light diffuser is illuminated with different spot sizes. For instance, it was reported in^59^ that the speckle size increases as the illuminated area decreases, regardless of the complex structure embedded in the phase and amplitude distribution of the beam illuminating the diffuser. Thus, if the CCD analysis window is considered to have a fixed area, increasing the speckle size results in a pattern with fewer speckles, but exhibiting a higher contrast $\left({g}_{self, max}^{\left(2\right)}>2.0\right)$. Conversely, large illumination diffuser areas lead to the construction of a pattern with a large number of speckles, with smaller sizes, resulting in a more homogeneous intensity distribution, i.e.*,* lower intensity contrast $\left({g}_{self, max}^{\left(2\right)}<2.0\right)$. For this reason, the IC-scan curves present a peak-to-valley structure opposite to those of D4σ, which directly measure the beam size in the detection plane.

To validate the experimental IC-scan results, numerical simulations were performed using the ${n}_{2}^{ethanol}=-2.2\times 1{0}^{-8} {\text{cm}}^{2}/{\text{W}}$ and $m=0.1$, which coincides with the values obtained by D4σ (also being close to those of Z-scan). The solid lines in Fig. 4c reveal the good agreement between the experimental results and the numerical simulation of well-characterized transparent NL media.

In a simpler way, the NL refractive index in IC-scan can also be obtained by using an *external reference method*, provided that the NL parameters ($m$ and ${n}_{2}$) of a reference material are known. For instance, Fig. 4d shows the evolution of the peak-to-valley variations of ${g}_{self}^{\left(2\right)}$, i.e. $\Delta {g}_{self, max}^{\left(2\right)}$, as a function of the incident intensity for pure ethanol. A linear behavior with slope ${S}_{ethanol}=\left(2.55\pm 0.05\right)\times {10}^{-2}{\text{cm}}^{2}/{\text{kW}}$ is obtained for intensities up to 42 kW/cm^2^. Assuming that ${n}_{2}$ is known for pure ethanol (reference material), similar to the Z-scan and D4σ techniques, where the peak-to-valley transmittance variations: $\Delta {T}^{p-v}\propto \Delta {\phi }^{NL}$ and the peak-to-valley second moment variations: $\Delta {m}_{2}^{p-v}\propto \Delta {\phi }^{NL}$, respectively, in IC-scan we propose that $\Delta {g}_{self, max}^{\left(2\right)}\propto \Delta {\phi }^{NL}=k{n}_{2}{L}_{eff}I$. Thus, the NL refractive index for a different material can be obtained by using the relationship: ${n}_{2}^{j}=\left({S}_{j}/{S}_{ref}\right){n}_{2}^{ref}$, where the subscripts *ref* and *j* represent the reference and the new NL media, respectively. This proposal was applied to the study of the NL refraction of pure methanol, which also exhibits a linear dependence of $\Delta {g}_{self, max}^{\left(2\right)}$ on the incident intensity (see Fig. 2S). By using ${S}_{methanol}=\left(3.60\pm 0.04\right)\times {10}^{-2}{\text{cm}}^{2}/{\text{kW}}$, calculated from the linear fit of the experimental results, and the ${n}_{2}^{ethanol}$ measured by IC-scan, it is possible to find ${n}_{2}^{methanol}=-\left(3.1\pm 0.3\right)\times 1{0}^{-8} {\text{cm}}^{2}/{\text{W}}$, which is very close to the ${n}_{2}$ values obtained by Z-scan $\left(-\left(3.3\pm 0.2\right)\times 1{0}^{-8} {\text{cm}}^{2}/{\text{W}}\right)$ and D4σ $\left(-\left(2.7\pm 0.4\right)\times 1{0}^{-8} {\text{cm}}^{2}/{\text{W}}\right)$, demonstrating the reliability of the IC-scan technique. The ${n}_{2}^{methanol}$ calculated by the *external reference method* coincides with the value obtained from the numerical fit $-\left(3.0\pm 0.2\right)\times 1{0}^{-8} {\text{cm}}^{2}/{\text{W}}$ described in the Numerical simulation section (see Fig. 2S of the supplementary material).

It is important to mention that the expression to calculate ${n}_{2}^{j}$, using the *external reference method*, is modified by a multiplicative factor when the reference and new NL material do not present the same nonlocality factor, *m*. In this work, the dependence of $\Delta {g}_{self, max}^{\left(2\right)}$ on *m* can be calculated numerically, as was done in previous works for Z-scan^52,53^. However, new studies are being developed to analytically describe IC-scan curves.

### NL refractive optical characterization of turbid media

**Figure 5:**
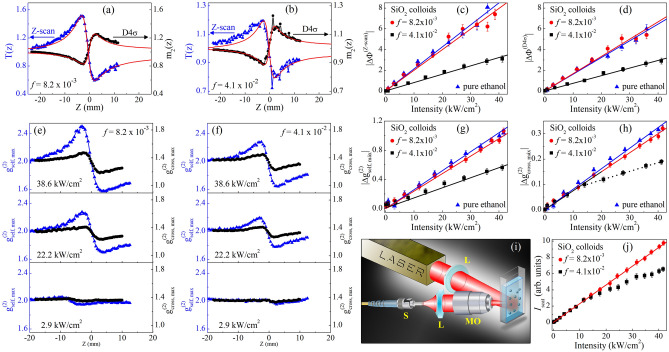
CA Z-scan and D4σ curves for colloids containing SiO_2_ NPs suspended in ethanol with (a) *f* = 8.2 × 10^–3^ and (b) *f* = 4.1 × 10^–2^, for an intensity of 22.2 kW/cm^2^. The solid curves in (**a**,**b**) were obtained by using the nonlocal nonlinearity model. (c, d) Intensity dependence of NL phase shift obtained from (**c**) CA Z-scan and (**d**) D4σ measurements. (**e**,**f**) IC-scan curves for SiO_2_ colloids obtained from 2D spatial intensity self-correlation (blue curves) and cross-correlation (black curves) functions and (**g**,**h**) their respective $\Delta {g}_{ max}^{\left(2\right)}$ versus incident intensity. (**i**) Experimental scheme and (**j**) result of the measurement of the scattered light intensity (*I*_scat_) as a function of the incident intensity for SiO_2_ colloids.

Although IC-scan can be used to characterize the NL refractive response of transparent media, the advantages of IC-scan over other techniques become relevant when measuring the NL response of scattering media. First, colloids containing SiO_2_ NPs suspended in ethanol were prepared as described in the NL media section. Since Rayleigh scattering is more predominant in the blue spectral region, large NP’s volume fractions were needed to induce from weak to moderate scattering at 788 nm. For instance, Fig. 5a illustrates a good signal-to-noise ratio in the CA Z-scan and D4σ curves for *f* = 8.2 × 10^–3^ and *I* = 22.2 kW/cm^2^. The experimental curves were fitted using the nonlocal nonlinearity model, obtaining as result a NL refractive index that coincides with that of pure ethanol $\left({n}_{2}^{ethanol}\right)$. The analysis indicates that, under the excitation conditions used here, SiO_2_ NPs play the role of light scatterers with negligible nonlinearity, i.e. ${n}_{2}^{Si{O}_{2}-colloid}=\left(1-f\right){n}_{2}^{ethanol}=99\%\left({n}_{2}^{ethanol}\right)$.

In addition, the experimental curves show that the higher the NPs concentration (*f* = 4.1 × 10^–2^), the lower the signal-to-noise ratio and, consequently, the greater the deviation against the theoretical (without dispersion) model, as shown in Fig. 5b. A compilation of the effective NL phase shift, $\Delta {\phi }_{eff}^{NL}$, measured by CA Z-scan and D4σ for pure ethanol and colloids containing SiO_2_ NPs can be seen in Fig. 5c,d, respectively. Notice, in both techniques, that $\Delta {\phi }_{eff}^{NL}$ for pure ethanol and the colloid with *f* = 8.2 × 10^–3^ are very close to each other, in agreement with Fig. 5a, for intensities up to 40 kW/cm^2^. Nevertheless, for *f* = 4.1 × 10^–2^, the slope of the $\Delta {\phi }_{eff}^{NL}$ curve as a function of $I$ for CA Z-scan (D4σ) is 2.6 (2.2) times less than that of pure ethanol, contradicting what is expected through ${n}_{2}^{Si{O}_{2}-colloid}=\left(1-f\right){n}_{2}^{ethanol}=96\%\left({n}_{2}^{ethanol}\right)$. In the latter case, it is evident that the scattering is responsible for causing distortions in the profile of the CA Z-scan and D4σ curves, leading to inadequate measurements of the NL refractive index in turbid media. Table 1 shows the ${n}_{2}^{Si{O}_{2}-colloid}$ obtained for both techniques using the nonlocal nonlinearity model.

**Table 1:** NL refractive indices measured for ethanol and SiO_2_ colloids, with different volume fractions (*f*), using Z-scan, D4σ and IC-scan techniques.

NL optical technique		${n}_{2}^{ethanol}$(cm^2^/W)	${n}_{2}^{Si{O}_{2}-colloid}$(cm^2^/W)	
			*f* = 8.2 × 10^–3^	4.1 × 10^–3^
Z-scan		−2.8 × 10^–8^	−2.6 × 10^–8^	−1.1 × 10^–8^
D4σ		−2.2 × 10^–8^	−2.2 × 10^–8^	−1.0 × 10^–8^
IC-scan	Self-correlation	−2.2 × 10^–8^	−2.1 × 10^–8^	−1.3 × 10^–8^
	Cross-correlation		−2.1 × 10^–8^	−2.1 × 10^–8†^

^†^NL refractive index measured for intensities up to 15 kW/cm^2^.

On the other hand, the IC-scan curves show greater robustness against the scattering caused by SiO_2_ NPs, even for larger concentrations, as shown in Fig. 5e,f. For visualization purposes, all IC-scan curves were set to start at ${g}_{max}^{(2)}\left(z=-20 {\text{mm}}\right)=2.0$, although the curves show a decrease in the maxima of the correlation functions due to contrast decrease caused by particle-induced light scattering. The vertical shift does not modify the results obtained since the measurement of ${n}_{2}^{Si{O}_{2}-colloid}$ by using the *external reference method* is based on the analysis of the intensity dependence of $\Delta {g}_{self, max}^{\left(2\right)}$. Figure 5g shows that the slope of the $\Delta {g}_{self, max}^{\left(2\right)}$ curves for the different concentrations of SiO_2_ NPs behaves in a similar way to that observed in $\Delta {\phi }_{eff}^{NL}$ for Z-scan and D4σ. As expected, similar values of ${n}_{2}$ were found for pure ethanol and the colloid with *f* = 8.2 × 10^–3^. Meanwhile, for the colloid with *f* = 4.1 × 10^–2^, the ${n}_{2}^{Si{O}_{2}-colloid}$ was 1.7 times lower than that of pure ethanol, as reported in Table 1. Although the ${n}_{2}^{Si{O}_{2}-colloid}$ obtained by IC-scan for the largest concentrations differs from the expected theoretical value $\left(96\%{n}_{2}^{ethanol}\right)$, its accuracy is higher than that of the Z-scan and D4σ techniques.

An adaptation in the analysis methodology of the speckle patterns captured by the CCD, in the IC-scan technique, allows to achieve more exact measurements of the NL refractive index in strong-scattering media. For this purpose, instead of the self-correlation function, it is proposed to analyze the 2D spatial intensity cross-correlation function $\left({g}_{cross}^{\left(2\right)}\left(\Delta r\right)=\frac{\langle \int {d}^{2}r{I}_{1}\left(r\right){I}_{2}\left(r+\Delta r\right)\rangle }{\int {d}^{2}r\langle {I}_{1}\left(r\right)\rangle \langle {I}_{2}\left(r+\Delta r\right)\rangle }\right)$ between the far-field intensity transverse profiles (*I*_1_ and *I*_2_) induced in two different regimes. The first regime is excited at low incident intensities (*I* = 0.1 kW/cm^2^), where linear scattering effects exist but refractive nonlinearities are negligible. Meanwhile, in the second regime, the incident intensities (*I* > 1.0 kW/cm^2^) are high enough to excite both linear and NL effects. Therefore, the cross-correlation function allows to analyze the statistical properties of the speckle patterns that were modified only by NL refraction effects.

The black curves in Fig. 5e,f show the new IC-scan profiles for SiO_2_ colloids obtained by analyzing the maximum values of the ${g}_{cross}^{\left(2\right)}$ as a function of the sample position. It is important to mention that the 2D spatial intensity cross-correlation function was also calculated from 50 consecutive images captured for *I*_1_ (linear regime) and *I*_2_ (NL regime). Notice from Fig. 5h that the $\Delta {g}_{cross, max}^{\left(2\right)}$ between the peak and the valley for pure ethanol and the SiO_2_ colloid with *f* = 8.2 × 10^–3^ evolve in a similar way with the increase of the incident intensity, in accordance with the other techniques. Even more interesting is that for intensities up to 15 kW/cm^2^, the IC-scan technique using the cross-correlation function is the only methodology that, as expected, shows that the slope of the $\Delta {g}_{cross, max}^{\left(2\right)}$ curve for the colloid with *f* = 4.1 × 10^–2^ is close to that of pure ethanol. As a result, ${n}_{2}^{Si{O}_{2}-colloid}=-\left(2.1\pm 0.1\right)\times 1{0}^{-8} {\text{cm}}^{2}/{\text{W}}$ is obtained for the most concentrated SiO_2_-colloid, corresponding to ~ 96% of the value obtained for pure ethanol, as indicated in Table 1. The results reveal the potential of the IC-scan technique to remove the contribution of linear scattering in the analysis of intensity cross-correlations, allowing a correct measurement of the NL refractive index in turbid media.

For *I* > 15 kW/cm^2^, it is observed that for the colloid with *f* = 4.1 × 10^–2^, $\Delta {g}_{cross, max}^{\left(2\right)}$ also deviates significantly from the values found for pure ethanol, indicating the contribution of some new NL phenomenon that influences the characterization of the NL refractive behavior. To understand the origin of the change in the slope of the $\Delta {g}_{cross, max}^{\left(2\right)}$ versus *I* curve, experiments to characterize the behavior of the scattered light intensity with the increase of the laser intensity were performed. In these experiments, a cell with 1.0 mm thickness, containing SiO_2_ colloids, was located in the focus of a 10 cm lens, identical to that used in the Z-scan, D4σ and IC-scan experiments. The scattered light was collected in a direction nearly perpendicular to the propagation direction of the incident laser beam by using a microscope objective, a plano-convex lens and a photodetector, as schematized in Fig. 5i.

Figure 5j shows the dependence of the scattered light intensity (at 788 nm) with the incident laser intensity for the SiO_2_ colloids. Notice that for *f* = 8.2 × 10^–3^, the scattered light intensity (*I*_scat_) presents a linear behavior (red line) versus the incident intensity that extends up to ~ 40 kW/cm^2^. However, similar to Fig. 5h for *f* = 4.1 × 10^–2^, *I*_scat_ exhibits a significant deviation from the linear behavior for *I* > 15 kW/cm^2^. This NL scattering contributions can be understood from the Rayleigh-Gans model^60^, by expressing the scattering coefficient as: ${\alpha }_{scat}={g}_{s}{\left(\Delta n\right)}^{2}$, where $\Delta n$ represents the difference between the effective refractive indices of the NP and the host medium, and ${g}_{s}$ is an intensity-independent parameter, but depends on the size, shape and concentration of the NPs and the optical wavelength. By considering the NL refractive behavior of the colloids $\left(\Delta n=\Delta {n}^{L}+\Delta {n}_{2}^{eff}I\right)$, it is possible to find expressions for the linear $\left({\alpha }_{scat}^{L}={g}_{s}{\left[{\Delta n}_{L}\right]}^{2}\right)$ and NL $\left({\alpha }_{scat}^{NL}=2{g}_{s}{\Delta n}_{L}{\Delta n}_{2}\right)$ scattering coefficients, with ${\alpha }_{scat}={\alpha }_{scat}^{L}+{\alpha }_{scat}^{NL}I$. Since the NL contribution of the SiO_2_ NPs was considered small compared to the solvent, ${\Delta n}_{2}$ corresponds mainly to the NL refractive index of ethanol, which became significant for higher intensities. Thus, as shown in Table 1, ${\alpha }_{scat}^{NL}<0$, decreasing the linear scattering coefficient for high intensities and corroborating the results of Fig. 5h,j. Therefore, in addition to the IC-scan technique allowing scattering-free NL refraction measurements, it also has the ability to distinguish linear and NL scattering contributions.

**Figure 6:**
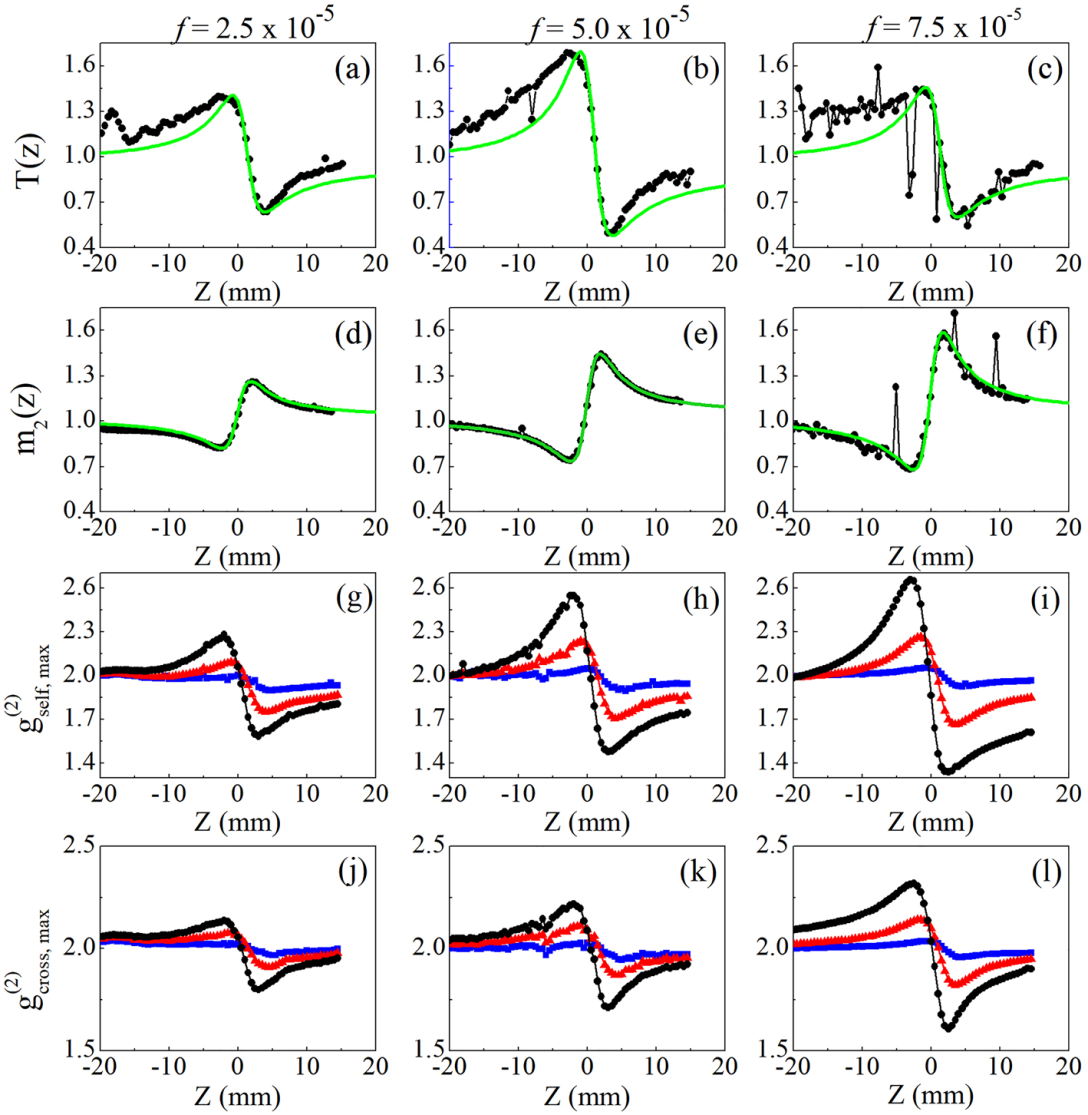
Normalized (**a**–**c**) CA Z-scan, (**d**–**f**) D4σ and (g-l) IC-scan curves for Au-NR colloids obtained from 2D spatial intensity (**g**–**i**) self-correlation and (**j**–**l**) cross-correlation functions, for Au-NRs colloids with volume fractions of (**a**,**d**,**g**,**j**) 2.5 × 10^–5^, (**b**,**e**,**h**,**k**) 5.0 × 10^–5^, (**c**,**f**,**i**,**l**) 7.5 × 10^–5^ and incident intensities of 0.1 kW/cm^2^ (blue squares), 0.5 kW/cm^2^ (red triangles) and 1.1 kW/cm^2^ (black circles). The solid (green) lines in (a-f) represent the best fit using the nonlocal nonlinearity model. For presentation purposes, all IC-scan curves have been shifted vertically to start at ${g}_{max}^{(2)}=2.0$.

A similar study was performed with Au-NRs colloids, where the nonlinearity is dominated by the thermal response of the nanoparticles. However, due to the NR’s dimensions, a relevant contribution from linear scattering is present in the *l*-LSP band^61^, which makes the Au-NRs behave as both scatterers and NL particles. This dual behavior of the Au-NRs makes its NL characterization, using the Z-scan technique, suffer from scattering-induced wavefront distortions that cause erroneous NL refractive index measurements. In fact, Fig. 6a–c exhibit CA Z-scan curves whose signal-to-noise ratio decreases drastically with increasing volume fraction. An even more critical result is that ${\Delta T}_{p-v}^{Z-scan}$ for the Au-NRs colloid with *f* = 7.5 × 10^–5^ is less than for the more dilute colloids. By fitting the experimental curves using the nonlocal nonlinearity model (green solid lines), NL refractive indices that do not obey monotonic growth as a function of Au-NR’s concentration are obtained, as shown in Table 2. Thus, the NL characterization of these scattering media, by using the Z-scan technique, contradicts the expected optical behavior in effective media theories whose nonlinearity is dominated by the NP’s response. A clear example is the Maxwell–Garnett theory^50,62^, where the effective third-order susceptibility is the result of the contributions of the host medium and the NPs susceptibilities, weighted through the volume fraction. Figures 6d–f show the D4σ curves for Au-NR colloids where, despite exhibiting a better signal-to-noise ratio, $\Delta {m}_{2}^{p-v}$ also does not grow proportionally to the increase in Au-NR concentration.

**Table 2:** NL refractive indices measured for Au-NR colloids using Z-scan, D4σ and IC-scan techniques.

NL optical technique		${n}_{2}^{AuNR-colloid}$(× 10^–6^ cm^2^/W)		
		*f* = 2.5 × 10^–5^	5.0 × 10^–5^	7.5 × 10^–5^
Z-scan		−0.56	−0.86	−0.75
D4σ		−0.27	−0.45	−0.46
IC-scan	Self-correlation	−0.56	−0.88	−1.04
	Cross-correlation	−0.86	−1.26	−1.73

**Figure 7:**
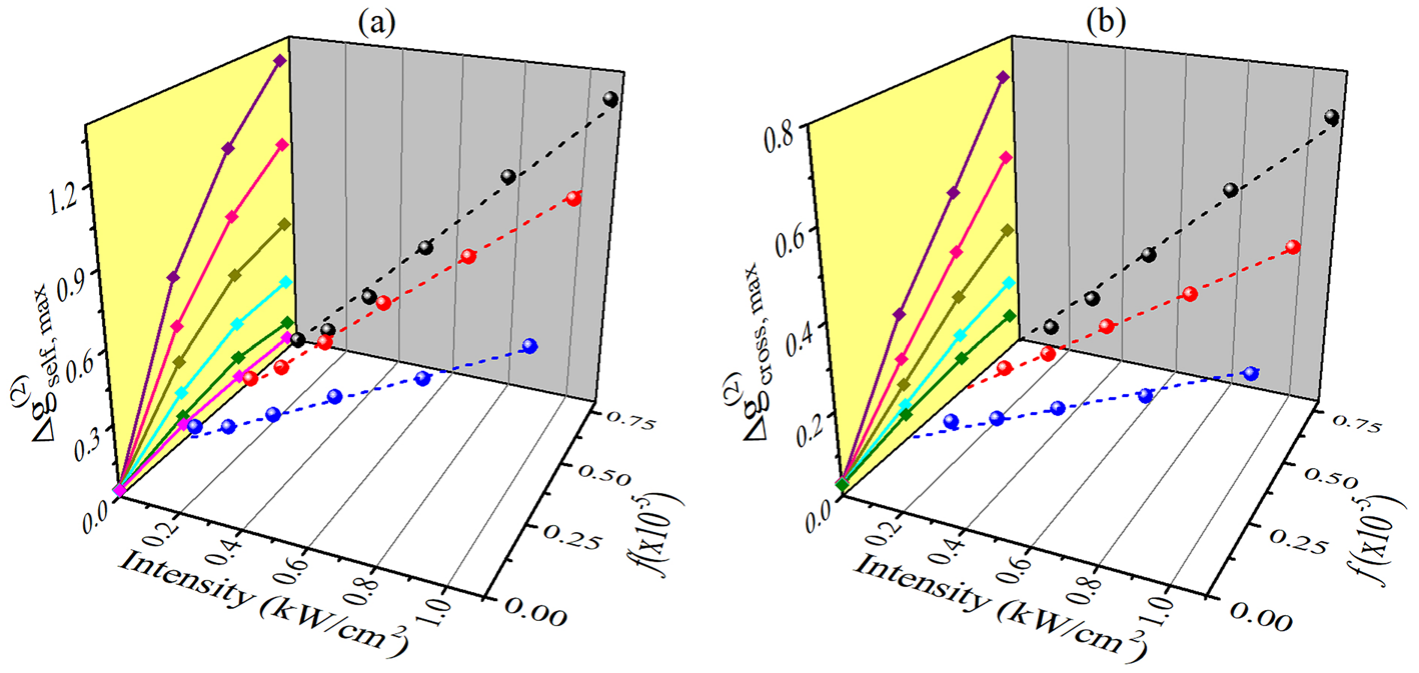
Intensity and volume fraction dependence of peak-to-valley variation of IC-scan curves obtained for Au-NRs colloids using the 2D spatial intensity (**a**) self-correlation and (**b**) cross-correlation functions. In the yellow planes, the curves were built from the projection of the values of $\Delta {g}_{self,max}^{\left(2\right)}$ and $\Delta {g}_{cross,max}^{\left(2\right)}$ versus *f*, for the different intensities. From top to bottom: *I* = 1.1, 0.8, 0.5, 0.3, 0.2 and 0.04 kW/cm^2^, the latter corresponding to the linear regime (used as a reference in the cross-correlation functions).

In contrast, the IC-scan curves, obtained through the analysis of the self- (Fig. 6g–i) and cross-correlation (Fig. 6j–l) functions, show excellent signal-to-noise ratios for all concentrations explored in this work. Furthermore, Fig. 7 exhibits a monotonic linear increase of $\Delta {g}_{max}^{\left(2\right)}\propto \Delta {\phi }^{NL}=k{n}_{2}{L}_{eff}I$ with incident intensity, as expected for both IC-scan configurations. Regarding the concentration dependence of the NL refractive index, measurements using the CA Z-scan (Fig. 6a–c) or self-correlation IC-scan (Fig. 7a) show that for larger volume fractions, $\Delta {\phi }^{NL}$ decreases or saturates, respectively, due to strong scattering. Nevertheless, when the IC-scan technique is applied using the cross-correlation functions (Fig. 7b), $\Delta {g}_{cross,max}^{\left(2\right)}$ presents a linear behavior with the NP’s volumetric fraction, preserving the validity of models such as the Maxwell–Garnett one to study the NL response of composite media. Therefore, the studies with Au-NR’s colloids reinforce the potential of the cross-correlation IC-scan technique to measure the NL refractive indices of turbid media.

## Conclusions

In summary, the present experiments demonstrate the potential of the IC-scan technique to adequately characterize the NL optical response of heterogeneous media, which can exhibit such strong (multiple) scattering that conventional NL spectroscopy techniques fail. The ability for NL characterization of turbid media with IC-scan lies in the analysis of the wavefront changes induced by the self-focusing and self-defocusing effects through the statistical properties of the speckle patterns that are formed in media with a high degree of scattering (or passing through an external light diffuser). Therefore, elastic scattering, which is detrimental to Z-scan and D4σ techniques, is the fundamental phenomenon that gives rise to the IC-scan technique. As a proof of principle, the NL refractive indices of colloids containing highly concentrated silica nanospheres in ethanol and gold nanorods in water were measured with Z-scan, D4σ and IC-scan. The results reveal that the IC-scan experiments present curves with a better signal-to-noise ratio, resulting in more precise measurements, as well as NL refractive index that are in agreement with the expected theoretical values. The accuracy of IC-scan measurements in highly scattering media is achieved by analyzing the cross-correlation functions between linear and NL regimes, which measure the influence of NL refraction on the generated speckle patterns, without the interference effects caused by linear scattering. In this way, IC-scan is presented as a powerful tool to characterize the NL refractive index of media with significant linear (elastic) scattering, such as inhomogeneous vitreous materials, biological media and liquid crystals, maintaining a simple experimental setup compared to other techniques. In addition, the IC-scan technique presented the ability to identify regions where NL scattering effects are relevant, which is a topic of considerable fundamental interest, and its study is currently in progress. New studies are also being carried out to characterize the NL response of liquid crystals, which have the ability to generate speckle patterns by themselves, configuring an adaptation to the IC-scan technique in which the light diffuser is dispensable.

## Supplementary Information

Supplementary Information.

## Data Availability

Data underlying the results presented in this paper are not publicly available at this time but may be obtained from the authors upon reasonable request.
