# Author Correction: Intensity correlation scan (IC-scan) technique to characterize the optical nonlinearities of scattering media

**DOI:** 10.1038/s41598-023-34978-z

**Published:** 2023-05-17

**Authors:** Mariana J. B. Crispim, Cícera C. S. Pereira, Nathália T. C. Oliveira, Martine Chevrollier, Rafael A. de Oliveira, Weliton S. Martins, Albert S. Reyna

**Affiliations:** 1 Programa de Pós-Graduação em Engenharia Física, Unidade Acadêmica do Cabo de Santo Agostinho, Universidade Federal Rural de Pernambuco, Cabo de Santo Agostinho, Pernambuco 54518-430 Brazil; 2 Programa de Pós-Graduação em Ciência de Materiais, Universidade Federal de Pernambuco, Recife, Pernambuco 50740-560 Brazil

Correction to: *Scientific Reports* 10.1038/s41598-023-34486-0, published online 04 May 2023

The original version of this Article contained an error in the Funding section.

"Conselho Nacional de Desenvolvimento Científico e Tecnológico (CNPq) - Universal 408016/2018-3; Fundação de Amparo à Ciência e Tecnologia do Estado de Pernambuco (FACEPE) - APQ-0962-1.05/21; Coordenação de Aperfeiçoamento de Pessoal de Nível Superior (CAPES)."

now reads:

"Conselho Nacional de Desenvolvimento Científico e Tecnológico (CNPq) - Universal 408016/2018-3; Fundação de Amparo à Ciência eTecnologia do Estado de Pernambuco (FACEPE) - APQ-0962-1.05/21 and APQ-1006-1.05/21; Coordenação de Aperfeiçoamento de Pessoal de Nível Superior (CAPES)."

The original Article has been corrected.

