# Supplementary material for: Intensity correlation scan (IC-scan) technique to characterize the optical nonlinearities of scattering media
Source: Sci Rep. 2023 May 4;13:7239. doi: 10.1038/s41598-023-34486-0 (PMC10160117; doi:10.1038/s41598-023-34486-0)
Supplement: Supplementary file 1 — Supplementary Information. [file 41598_2023_34486_MOESM1_ESM.pdf]

SUPPLEMENTARY MATERIAL

Intensity correlation scan (IC-scan) technique to characterize the optical nonlinearities of scattering media

Mariana J. B. Crispim[1], Cícera C. S. Pereira[1], Nathália T. C. Oliveira[2], Martine Chevrollier[1], Rafael A. de Oliveira[1], Weliton S. Martins[1], and Albert S. Reyna[1,*]

[1]Programa de Pós-Graduação em Engenharia Física, Unidade Acadêmica do Cabo de Santo Agostinho, Universidade Federal Rural de Pernambuco, Cabo de Santo Agostinho, Pernambuco 54518-430, Brazil
[2]Programa de Pós-Graduação em Ciência de Materiais, Universidade Federal de Pernambuco, Recife, Pernambuco 50740-560, Brazil
[*]areynao@yahoo.com.br

1. Thermal effects in Z-scan and D4σ through local and nonlocal nonlinearity models

Figure 1S shows the comparison between local and nonlocal models to fit the experimental Z-scan and D4σ curves obtained for ethanol by using a high-repetition-rate femtosecond Ti:sapphire laser. For demonstration purposes, the blue curves show curve fitting, using the numerical simulation described in main text, but considering the local model (m = 2.0), as described by Sheik-Bahae *et al.* [1] and Amaral *et al.* [2]. Nonlinear refractive indices of -11.0×10^{-9} cm^2/W (Z-scan) and -4.9×10^{-9} cm^2/W (D4σ) were obtained for this analysis. Although the value obtained by D4σ (Z-scan) is very close (of the same order of magnitude) to that recently reported in the literature ($n_2 = -4.0 \times 10^{-9}$ cm^2/W) [3], the fit does not adequately describe the experimental results. On the contrary, considering that a quasi-continuous pulse excitation causes cumulative thermal effects, the experimental measurements were also fitted using the nonlocal model. The red lines show the best fits using $m = 0.1$ and $n_2 = -2.8 \times 10^{-8}$ cm^2/W ($n_2 = -2.2 \times 10^{-8}$ cm^2/W) for Z-scan (D4σ) technique. Therefore, in this work the nonlocal model was used since it more adequately simulates the experimental results.

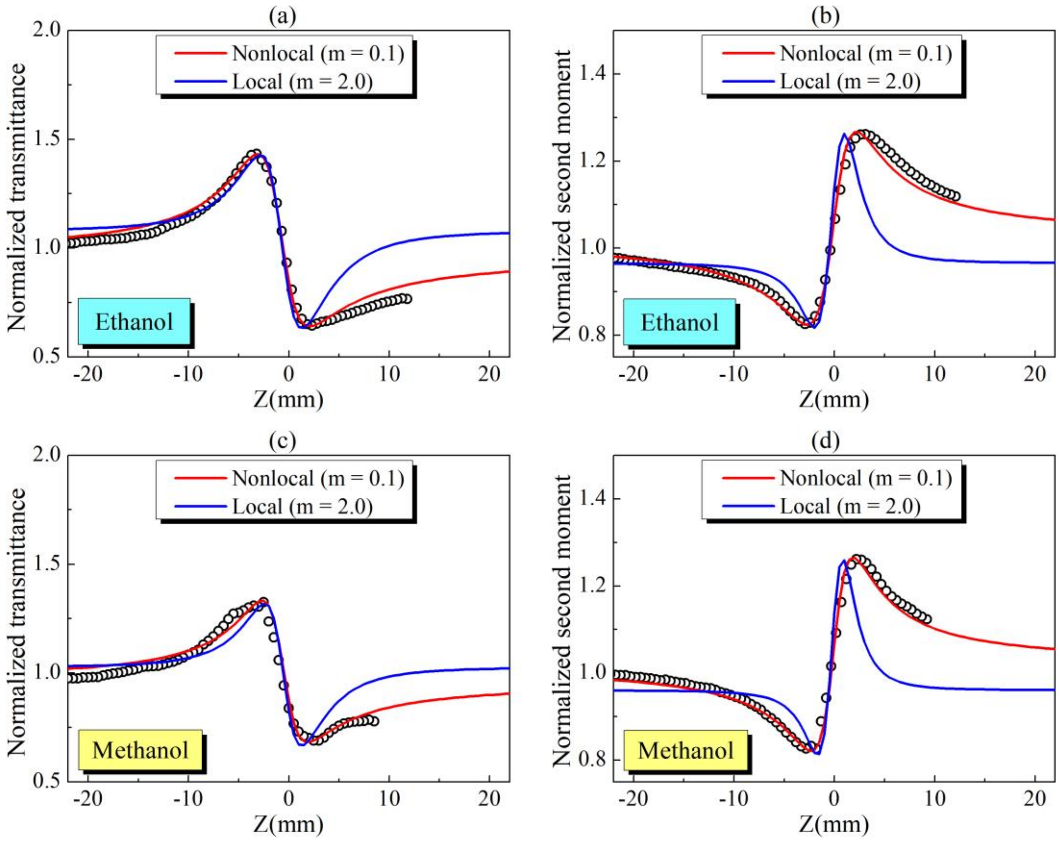

Fig. 1S: Experimental (a, c) Z-scan and (b, d) D4σ curves for (a, b) ethanol and (c, d) methanol, as well as their respective fits using the local (blue lines) and nonlocal (red lines) models.

A similar analysis was performed for methanol [see Fig. 1S(c) and Fig. 1S(d)], showing that the nonlocal model more adequately describes the experimental results under the conditions used in this work. Nonlinear refractive indices of -3.5×10^{-8} cm^2/W (-3.0×10^{-8} cm^2/W) and -5.9×10^{-9} cm^2/W (-3.2×10^{-8} cm^2/W) were obtained from the Z-scan and D4σ techniques, respectively, using the local (nonlocal) model.

2. IC-scan curves for methanol

Fig. 2S(a) shows the experimental IC-scan curves obtained for methanol by analyzing the maximum values of the self-correlation function at the different sample positions along the z-axis. For incident intensities used between 1.0 and 30 kW/cm^2, Fig. 2S(b) shows the linear behavior of the peak-to-valley variation of the IC-scan curves, $\Delta g^{(2)}_{self,max}$.

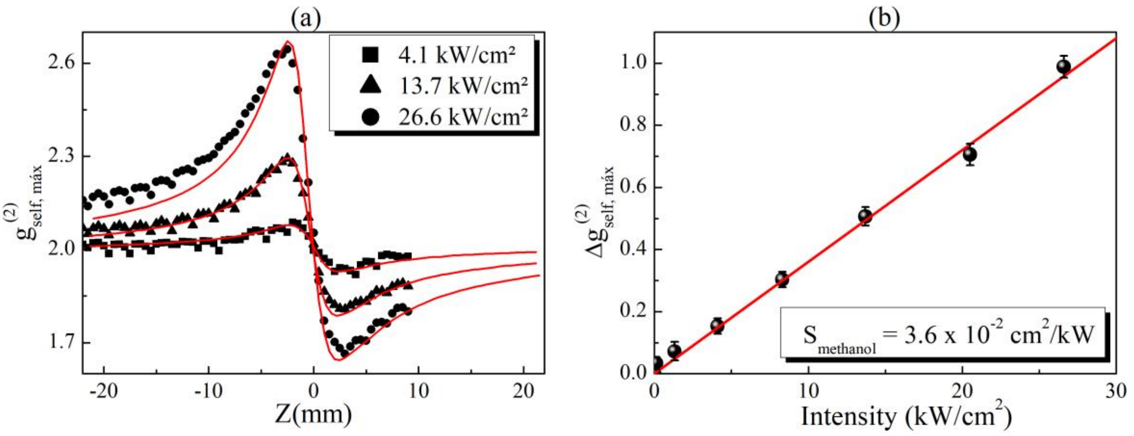

Fig. 2S: (a) Experimental and numerical (solid lines) IC-scan curves for methanol. (b) Peak-to-valley variation of IC-scan curves for methanol as a function of incident intensity.

The solid lines in Fig. 2S(a) represent the result of the simulation described in section 3 of the paper (main text), by using $n_2^{methanol} = -(3.0 \pm 0.2) \times 10^{-8}\ \mathrm{cm}^2/\mathrm{W}$, a value close to that measured by the *external reference method* $[-(3.1 \pm 0.3) \times 10^{-8}\ \mathrm{cm}^2/\mathrm{W}]$.

3. References

[1] M. Sheik-Bahae, A. A. Said, T. H. Wei, D. J. Hagan, and E. W. Van Stryland, "Sensitive measurements of optical nonlinearities using a single beam," IEEE J. Quantum Electron. **QE- 26**, 760-769 (1990).

[2] A. M. Amaral, H. A. Mejía, E. L. Falcão-Filho, and C. B. de Araújo, "D4σ curves described analytically through propagation analysis of transverse irradiance moments," Opt. Lett. **41**, 2081-2084 (2016).

[3] J. E. Q. Bautista, M. L. da Silva-Neto, C. L. A. V. Campos, M. Maldonado, C. B. de Araújo, and A. S. L. Gomes, "Thermal and non-thermal intensity dependent optical nonlinearities in ethanol at 800 nm, 1480 nm, and 1560 nm," J. Opt. Soc. Am. B **38**, 1104-1111 (2021).
